# Research progress of self-assembled nanogel and hybrid hydrogel systems based on pullulan derivatives

**DOI:** 10.1080/10717544.2018.1425776

**Published:** 2018-01-15

**Authors:** Tao Zhang, Ruyi Yang, Shengnan Yang, Jibin Guan, Dong Zhang, Yan Ma, Hongzhuo Liu

**Affiliations:** a School of Pharmacy, Shenyang Pharmaceutical University, Shenyang, China;; b School of Chinese Materia Medica, Guangzhou University of Chinese Medicine, Guangzhou, China

**Keywords:** Carrier, pullulan, self-assembled nanogel, hybrid hydrogel, research progress

## Abstract

Polymer nano-sized hydrogels (nanogels) as drug delivery carriers have been investigated over the last few decades. Pullulan, a nontoxic and nonimmunogenic hydrophilic polysaccharide derived from fermentation of black yeast like Aureobasidium pullulans with great biocompatibility and biodegradability, is one of the most attractive carriers for drug delivery systems. In this review, we describe the preparation, characterization, and ‘switch-on/off’ mechanism of typical pullulan self-assembled nanogels (self-nanogels), and then introduce the development of hybrid hydrogels that are numerous resources applied for regenerative medicine. A major section is used for biomedical applications of different nanogel systems based on modified pullulan, which exert smart stimuli-responses at ambient conditions such as charge, pH, temperature, light, and redox. Pullulan self-nanogels have found increasingly extensive application in protein delivery, tissue engineering, vaccine development, cancer therapy, and biological imaging. Functional groups are incorporated into self-nanogels and contribute to expressing desirable results such as targeting and modified release. Various molecules, especially insoluble or unstable drugs and encapsulated proteins, present improved solubility and bioavailability as well as reduced side effects when incorporated into self-nanogels. Finally, the advantages and disadvantages of pullulan self-nanogels will be analyzed accordingly, and the development of pullulan nanogel systems will be reviewed.

## Introduction

The use of nanodrug delivery systems has resulted in significant advances in reduced toxicity, modified release, and enhanced treatment efficiency (Mura et al., 2013). The development of nanocarriers, including nanoparticles, liposomes, vesicles, carbon nanotubes, polymeric micelles, nanoemulsions, microspheres, nanogels, and prodrug-nanosystems has provided alternative options for the prevention, treatment, and diagnosis of diseases (Pehlivan, 2013). Nanogels are endowed with unique physicochemical and biological properties that enable them to encapsulate and protect many payload drugs, confer high stability for prolonged circulation, and participate in versatile drug release protocols; additionally, they possess stimuli-responsive behavior potential, tunable size, and can assist with facile removal of empty devices (Maya et al., 2013). Hence, there has been enormous interest in nanogel systems (Varshosaz et al., 2017).

**Figure 1.**
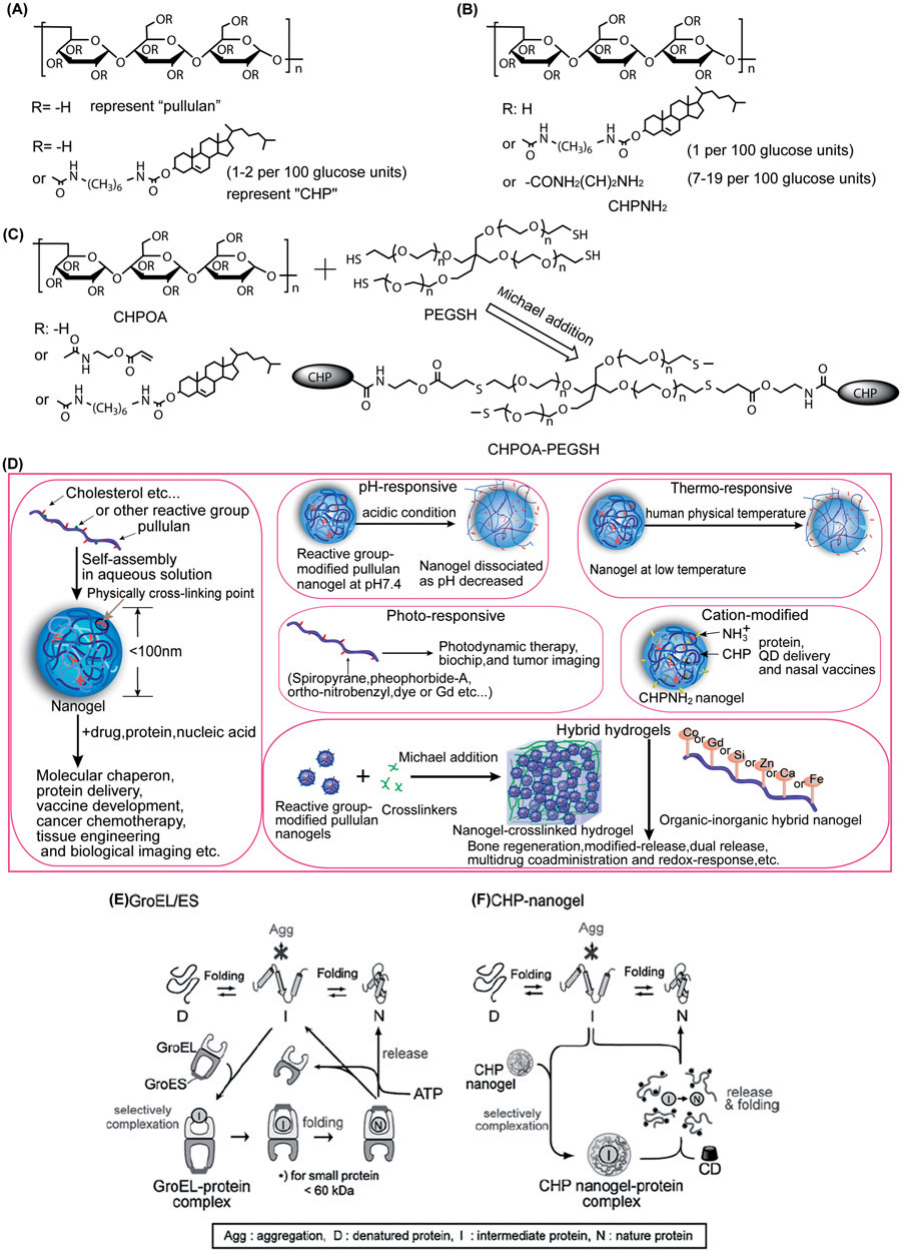
Chemical structures of (A) pullulan, (A) cholesterol-bearing pullulan (CHP), (B) CHPNH2, and (C) CHPOA-PEGSH; (D) Pullulan-based nanogels and hybrid hydrogels; and (E, F) schematic representation of chaperone-assisted refolding.

Nanogel is a gel with a nonfluid colloidal/polymer network but a diameter less than 100 nm (Tahara & Akiyoshi, 2015). Because of their hydrophilic three-dimensional macromolecular networks, polymer nanogels have been extensively studied as functional materials in biotechnological and biomedical fields (Zhang et al., 2015; Yahyaei et al., 2017). Similar to other polysaccharides such as chitosan (Huang & Lapitsky, 2017), hyaluronic acid (Wei et al., 2013), mannan (Ferreira et al., 2010), cycloamylose (Tahara et al., 2015), dextrin (Molinos et al., 2012), and enzymatically synthesized glucogen (Takeda et al., 2013), pullulan also has played a critical role in nanogel systems in recent years (Sasaki & Akiyoshi, 2010; Chacko et al., 2012). Pullulan is one of the commercially emerging aqueous polysaccharides synthesized by the yeast-like fungus *Aureobasidium pullulans*. It consists of hundreds of repeated units of the maltotriose trimer α-d-glucopyranosyl-(1 → 6)-α-d-glucopyranosyl-(1 → 4)-α-d-glucopyranosyl-(1 → 4)- (Figure 1(A)). The relative molecular mass can reach more than 1 × 10^4^ Da (1 × 10^5^ Da and 2 × 10^5^ Da are most frequently used). Although many active sites of pullulan may be modified and grafted, some evidence has revealed that extrinsic groups preferably introduce at the C-6 hydroxyl groups of pullulan (Bruneel & Schacht, 1994).

Innumerable examples demonstrate that partial modification of water-soluble polymers affects the solution properties (Zhu et al., 2017). Furthermore, chemical derivatization can control pullulan’s solubility with reactive groups accordingly (Jung et al., 2003; Singh et al., 2008). If modified by a hydrophobe such as cholesterol, pullulan becomes an amphiphilic molecule in the form of cholesterol-bearing pullulan (CHP, Figure 1(A)), which could act as an excellent nanogel carrier (Akiyoshi et al., 1993). CHP nanogels present great biocompatibility and biodegradability, nontoxicity, ease of preparation, reversible connections, thermal stability, and suppression of massive aggregation of proteins (Ayame et al., 2008; Boridy et al., 2009; Sawada & Akiyoshi, 2010; Yuki et al., 2013; Wang et al., 2014). Additionally, pullulan or CHP can be modified by other pharmaceutical agents to achieve superior applications in the biomedical field (Ferreira et al., 2011; Nakahashi-Ouchida et al., 2017). The text below mainly introduces the origin, concept, mechanism, and application of major pullulan nanogels and related hybrid hydrogels and will assist researchers in understanding the development of the nanogel field and other relevant delivery systems.

## Concept and mechanism of typical pullulan nanogels

### Synthesis, preparation, and characterization of typical pullulan nanogels

The nanogel system based on CHP backbone is the most popular object of study, and accordingly, an introduction explaining cholesterol-modified pullulan is below. Other pullulan nanogels or reactive group-modified pullulan nanogels will be explained and analyzed in the specific chapter of this article when necessary.

Figure 1(A) illustrates the basic structures of pullulan and proportional CHP. CHP is synthesized by acylation between cholesterol and pullulan with N-(6-isocyanatohexyl) carbamate as a transition. Akiyoshi et al. discovered that CHP spontaneously formed a monodispersed spherical nanogel in water without adding any other adjuvants (Akiyoshi et al., 1993; Akiyoshi & Sunamoto, 1996). A single nanogel consisted of approximately 4-6 CHP macromolecules, and the contour length of a CHP molecule was at 44.59 ± 18.13 nm (Lee & Akiyoshi, 2004). The main driving force of amphiphilic nanogel formation is hydrophobic interaction. Fluorescence experiments suggested that CHP produces two domains, one consisting of a hydrophilic polysaccharide skeleton and the other domain consisting of the hydrophobic cholesterol moieties that form physical cross-links within the aggregate (Figure 1(D)). The size of the self-aggregates decrease with an increase in the degrees of substitution of the cholesteryl moiety (13.7 nm → 8.4 nm) (Akiyoshi et al., 1997). There are usually approximately 1–2 cholesterol molecules grafted to per 100 anhydroglucoside units of pullulan for colloidal stability (Akiyoshi et al., 1996). The critical aggregation concentration was very low (∼10 μg/ml). Additionally, the molecular weight (MW) and concentration of pullulan also influences the mechanical properties and gelation behavior of CHP nanogels. CHP nanogel made from 1.0 × 10^5^ or 4.0 × 10^5^ MW pullulans preferred to form a macrogel over 3.0 wt%, whereas CHP nanogel consisting of 0.55 × 10^5^ MW pullulan did not form a macrogel (Sekine et al., 2014). Recently, details regarding the spatial structure of CHP nanogels have been reported via contrast variation small-angle neutron scattering. The effective spherical radius of the skeleton formed by pullulan chains was found to be 8.1 nm, which is slightly smaller than that of the hydrodynamic (10 nm). There were approximately 19 cross-linking points, one of which was formed by the aggregation of 3.4 cholesterol groups in the CHP nanogel (Inomoto et al., 2009; Sekine et al., 2016).

### Mechanism of molecular capture and release fromCHP nanogels

As described above, hydrophobic interaction between cholesteryl groups of the nanomatrix and hydrophobic domain of the encapsulated protein is the primary capture motivation. Kinetic analysis suggested that a two-step process of capturing bovine serum albumin (BSA) occurs, where initially, there is the fast pre-equilibrium of looser binding of BSA to the self-nanogel followed by the slower process of tighter inclusion into the hydrogel network (Nishikawa et al., 1996). This illustrates that the stabilization of protein molecules within nanogel depends on molecular interactions, and when troubled in a segregated nanomatrix, the protein molecules will be more stable (Ayame et al., 2008).

Despite the architectural merits of CHP nanogels, it is a challenge to determine how to unload their cargo when they carry proteins into a living body. In general, molecular chaperones like heat shock protein (HSP, called GroEl/ES, Figure 1(E)) become involved in the procedure of intracellular protein folding in a living system (Horwich, 2014). Not only do the chaperones prevent aggregation from occurring, but they also permit proteins to maintain unfolded conformations or native activity, and additionally, they allow the assistance of ATP and another co-chaperone when necessary. Similarly to natural molecular chaperones, the nanogel chaperone system plays an essential role in the release of proteins. Akiyoshi put forward the concept of ‘artificial chaperone’ that interacts with CHP nanogels and also confirmed the chaperone-like mechanism (Asayama et al., 2008). It was thought that as a host, CHP nanogels prevented aggregation of denatured proteins or intermediate proteins upon refolding, while cyclodextrin (CD) served as the role of ATP in the formed inclusion complexes. After adding CD, by virtue of a hydrophobic cavity, CD could dissociate interactions between nanogels and proteins by vigorously combining with the hydrophobic cholesteryl groups of CHP (Gao et al., 2013), and then CHP nanogels were dissociated into individual CHP chains. Figure 1(F) illustrates that the folded (refolded) proteins were released in a native and active form (Nomura et al., 2003; Takahashi et al., 2015). Another explanation for the release of proteins is the protein-exchange mechanism, in which BSA, with its stronger hydrophobicity, is able to displace protein encapsulated from the nanogel (Shimoda et al., 2012a).

Different types and concentrations of CD can control the release rate of protein drugs. Although β-CD exhibits the highest refolding efficiency compared with α-CD and γ-CD, methyl-β-CD is even more useful (Gao et al., 2013). CHP nanogels are easily dissociated as the concentration of CD increases (Inomoto et al., 2009). However, all types of β-CD enable nanogels to dissociate entirely beyond a specific concentration (Nomura et al., 2005; Sawada et al., 2011). The optical interferometric technique indicated that methyl-β-CD induced the largest swelling changes, whereas α-CD led to the smallest swelling changes in the same CHP gels, arising from the capability of supermolecular self-assembly (Gao et al., 2013). Table 1 shows recent studies of CHP nanogels.

**Table 1.** Summary of recent studies of CHP nanogels.

Loaded objects	Types	Applications	References
Doxorubicin	Antitumor drug	Sustained-release	(Akiyoshi & Sunamoto, 1996)
Methacrylate random copolymer	Antimicrobial agent	Tunable activity	(Takahashi et al., 2015)
Prostaglandin E_1_	Anabolic agent	Wood healing	(Kobayashi et al., 2009)
Prostaglandin E_2_	Anabolic agent	Bone repair	(Kato et al., 2007)
Green fluorescent protein	Protein	Tunable release	(Asayama et al., 2008; Sasaki et al., 2010)
Bovine serum albumin	Protein	Tunable release	(Nishikawa et al., 1996)
Insulin	Protein	Enhanced stability	(Akiyoshi et al., 1998)
Oncogene erbB-2/neu/HER2	Protein	Induced cellular and humoral immune responses	(Gu et al., 1998)
amyloid β-protein	Protein	Alzheimer’s disease therapy	(Ikeda et al., 2006)
Truncated HER2 protein 1-146	Protein	Specific T cell immune responses	(Kitano et al., 2006)
NY-ESO-1 protein	Protein	T cell immunomonitoring and tumor responses	(Uenaka et al., 2007)
146HER2/Recombinant human granulocyte-macrophage colony-stimulating factor	Protein	Humoral immune responses	(Kageyama et al., 2008)
Melanoma antigen gene-A4 protein	Protein	Clinical cancer vaccination therapy	(Kyogoku et al., 2016)
Carbonic anhydrase B, Citrate synthase, Horseradish peroxidase	Enzyme	Molecular chaperone	(Nomura et al., 2005; Sawada et al., 2006, 2011)
α-Chymotrypsin	Enzyme	Tunable activity and thermal stability	(Nishikawa et al., 1994)
Lipase	Enzyme	Colloidal and thermal stabilization	(Sawada & Akiyoshi, 2010)
Synthetic long peptide antigen	Antigen	Antitumor vaccine of targeting macrophages	(Muraoka et al., 2014)
Interleukin-12	Cytokine	Sustained-release for tumor immune therapy	(Shimizu et al., 2008)
Tumor necrosis factor (TNF)-α	Cytokine	Protecting bone resorption	(Nagano et al., 2011)
Tumor necrosis factor (TNF)-α	Cytokine	Vaccine adjuvant against influenza virus	(Nagatomo et al., 2015)
9-mer HER2p63-71 peptide	Peptide	Tumor immune therapy	(Ikuta et al., 2002)
DNA	DNA	DNA separation	(Kondo et al., 2010)
Dex	Dye	Reduced cytotoxicity	(Gupta & Gupta, 2004)

## What are hybrid hydrogels?

Hybrid hydrogels can be defined as a complex composed of hundreds of chemical cross-linking nanogels. The porous (Yamane et al., 2008) or raspberry-like (Hasegawa et al., 2009) hydrogels showed high mechanical properties (Ma et al., 2017; Maeda et al., 2017), two well-defined networks such as a nanogel intranet work structure of less than 10 nm (physically cross-linking), and an internetwork structure of several hundred nanometers (chemical cross-linking) (Morimoto et al., 2005a) (Figure 1(C,D)). The network of hydrogels maintains stability depending on crosslinkers such as PEGSH. Furthermore, the well-dispersed nanogels still revealed high chaperone-like activity, and drugs were mostly trapped in the nanogel domains (Hirakura et al., 2010; Sekine et al., 2012). Nanogel crosslinked hydrogels that are produced via a bottom-up method are useful as novel scaffold materials for controlling drug release and preparing multi-functional nanoparticles (Sekine et al., 2012; Shimoda et al., 2012a,b; Tahara et al., 2013; Yokota et al., 2014; Hashimoto et al., 2015). Notably, hybrid hydrogels have promising applications in tissue engineering, especially regenerative medicine including bone regeneration (Kato et al., 2007; Hayashi et al., 2009; Kamolratanakul et al., 2011; Fujioka-Kobayashi et al., 2012; Shimoda et al., 2016). Table 2 describes recent studies of nanogel crosslinked hydrogels, of which cholesterol-bearing pullulan acryloyl modified–polyethylene glycol thiol modified (CHPOA-PEGSH), a representative composition, is illustrated in Figure 1(C).

**Table 2.** Summary of recent studies of nanogel crosslinked hydrogels.

Hybrid hydrogels	Nanogel/crosslinkers	Loaded objects	Applications	References
CHPOA-PEGSH	CHPOA: R: H or Cholesteryl group or **AOI**^(1)^; **PEGSH**^(2)^	Liposome	Dually controlled-release	(Sekine et al., 2012)
CHPOA-PEGSH		BMP2 protein, FGF18 protein	Multidrug delivery for bone repair	(Fujioka-Kobayashi et al., 2012)
CHPOA-PEGSH		Insulin	hydrolysis mechanism and exchange reactions	(Shimoda et al., 2012b)
CHPOA-PEGSH		sFGFR2^S525W^	cytokine therapy	(Yokota et al., 2014)
CHPOA-PEGSH		W9 peptide	Bone repair, sustained-release	(Sato et al., 2015)
CHPOA-PEGSH		Vitronectin, mouse embryo fibroblast cells	Artificial extracellular matrices	(Hashimoto et al., 2016)
CHPOA-PEGSH		Insulin	Longer elimination half-life	(Shimoda et al., 2012a)
CHPOA-PEGSH		Silicone sheet	Wound healing	(Maeda et al., 2017)
CHPA-PEGSH	CHPA: R: H or Cholesteryl group or − COCH = CH_2_; **PEGSH**^(2)^	Prostaglandin E_2_, BMPprotein, PDGF protein	Bone repair	(Kato et al., 2007; Hayashi et al., 2009; Kamolratanakul et al., 2011; Miyahara et al., 2012)
CHPA-PEGSH		Interleukin-12	Long-term stability	(Hasegawa et al., 2009)
CHPOA-PEGDA	CHPOA; **PEGDA**^(3)^	Insulin	Tunable activity	(Tahara et al., 2013)
CHPOA-P(MAA-g-EG)	CHPOA; P(MAA-g-EG)	Pregabalin	pH-responsive release	(Cinay et al., 2017)
CHPMA-MPC	CHPMA: R: H or Cholesteryl group or − COC(CH_3_)=CH_2_; **MPC**^(4)^	Insulin, Carbonicanhydrase B	Molecular chaperone	(Morimoto et al., 2005a,b)
CHP-HA	CHP: R: H or Cholesteryl group; **HA**^(5)^	Peptide-1, insulin, erythropoietin	Molecular chaperone, sustained-release	(Hirakura et al., 2010)
PHD	Pullulan-collagen; 1,2,7,8-diepoxyoctane	1,2,7,8-diepoxyoctane	Tissue filler materials	(Ma et al., 2017)

AOI: 2-(acryloyloxy)ethyl isocyanate; PEGSH: pentaerythritol tetra (mercaptoethyl)polyoxyethylene; PEGDA: polyrthylene glycol diacrylate; MPC: 2-methacryloyloxyethyl phosphorylcholine; HA: chemically cross-linked Haluronan.

Important structures marked with consecutive numbers (1)–(5) are redrawn within the Supplemental File ‘Numbered Structures’.

For instance, hyaluronan modified with 2-aminoethyl methacrylate, which was cross-linked via Michael addition in the presence of CHP nanogels (termed CHP-HA shown in Table 2), could spontaneously trap glucagon-like peptide-1, insulin, and human recombinant erythropoietin into the nanogel while hyaluronan was the biocompatible controlled release matrix outside (Hirakura et al., 2010). Recombinant human bone morphogenetic protein (BMP2) (or tumor necrosis factor receptor 2 (TNFR2)) was applied to a skull bone defect for bone repair by increasing the number of osteoblasts (Nagano et al., 2011; Fujioka-Kobayashi et al., 2012). Shimoda designed a novel hybrid hydrogel with acryloyl group-modified CHP (CHPOA) nanogels as crosslinking domains, which were incubated with PEGSH at 37 °C for 24 h under humidified conditions. The reason that sustained-release is used is because it enhances half-life due to the presence of crosslinker PEG chains, which prevent nonspecific protein absorption and exhibit slow hydrolysis kinetics (Shimoda et al., 2012b). CHPOA-PEGSH hydrogels for bone or skin repair could prolong the interval of prostaglandin E2 or prostaglandin E1, which are produced in the injection site against systemic effects (Kato et al., 2007). The combination of the CHPOA-PEGSH hydrogel with growth factors FGF18 and BMP2 strongly enhanced and stabilized BMP2-dependent bone repair (Fujioka-Kobayashi et al., 2012).

Multi-functional hybrid hydrogels revealed the potential of sequential dual release (Shimoda et al., 2012a) and multidrug co-administration (Sekine et al., 2012). For example, cholesterol moieties of CHP incorporated into liposomes through hydrophobic interaction and Figure 1(C) shows that the final formed hydrogels may be attributed to Michael addition between the acryloyl group of CHPOA and the thiol group of PEGSH. It has been proven that nanogel-trapped agents such as liposome (Sekine et al., 2012), insulin (Shimoda et al., 2012b), and mouse embryonic fibroblasts (Hashimoto et al., 2015) were released from the hydrogel by degradation of CHPOA-PEGSH hydrogels that occurred by hydrolysis of the ester bonds within the acryloyl group. Additionally, the release rate of nanogel accelerated along with increasing pH; the hydrogel was wholly degraded within 20 days at pH 8.0, which was two times faster than that of pH 6.0 (Shimoda et al., 2012b).

In addition to the graft of organic agents, combinations with inorganic substances may produce a novel nanomaterial (Sugawara et al., 2006; Yamane et al., 2008; Katagiri et al., 2013; Tsuchido et al., 2015). For instance, organic–inorganic hybrid nanogels with dual network structures were prepared by condensation of silanol groups grafted to CHP. They showed a narrow particle size distribution of approximately 90 nm in diameter, and colloidal stability in the presence of methyl-β-CD (Yamane et al., 2008). Another exciting development is that the coordination of zinc ions contributed to the successful preparation of protein nanogels using vitamin B6 (pyridoxal)-bearing pullulan as a bio-crosslinker and anionic bovine serum albumin (BSA) as a model protein (Tsuchido et al., 2015).

## Functionality of pullulan nanogels

According to these superior properties, investigators designed various functional pullulan nanogels such as those with cation-modified surface, stimulating sensitivity (pH-responsive, thermo-responsive, photo-responsive, and redox-responsive), modified-release and targeting properties. Therefore, these nanogels that entrapped drugs (paclitaxel, doxorubicin, carmofur, clonazepam, diphenhydramine, and pregabalin), proteins (enzymes, insulin, antigen, cytokines, peptides), and nucleic acids were used for cancer chemotherapy, protein delivery, and artificial vaccines. The nanogels could be combined with liposomes (Sekine et al., 2012; Hashimoto et al., 2015), emulsions (Akiyoshi & Sunamoto, 1996), particles (Sugawara et al., 2006; Shimoda et al., 2016), and even cells (Hashimoto et al., 2015, 2016) and then administered. Herein, we will demonstrate pullulan nanogel systems in detail in response to different functional classifications.

### Cation-modified

Cationic CHP (called cCHP or CHPNH2, shown in Figure 1(B) and Table 3) nanogels containing drugs were prepared by simple mixing and incubation (Hasegawa et al., 2005; Watanabe et al., 2011). In general, cCHP nanogels composed of a dozen ethylenediamine groups interact strongly with proteins, and this mechanism is the molecular basis for intracellular protein (Ayame et al., 2008; Boridy et al., 2009; Watanabe et al., 2011; Shimoda et al., 2014) or quantum dot (QD) (Hasegawa et al., 2005; Toita et al., 2008) delivery systems and nasal vaccines (Nochi et al., 2010; Kong et al., 2013; Yuki et al., 2013; Shimoda et al., 2014; Fukuyama et al., 2015).

**Table 3.** Summary of recent studies of responsive pullulan nanogels.

Nanogel	Typical structures	Loaded objects	Applications	References
CHPNH_2_	R: H or Cholesteryl group or − CONH(CH_2_)_2_NH_2_	Quantum dots, Bovine serum albumin	Live cell imaging	(Hasegawa et al., 2005; Toita et al., 2008)
CHPNH_2_		β-galactosidase, amyloid β-protein, green fluorescent protein, TAT-GFP protein, Bcl-xL protein	Intracelluar protein delivery, inhibition of Aβ aggregation, cancer therapy and immune regulation	(Ikeda et al., 2006; Ayame et al., 2008; Watanabe et al., 2011)
RGD-cCHP		Bovine serum albumin	Targeted protein delivery	(Shimoda et al., 2011)
cCHP		Pneumococcal surfaceprotein A	Nasal vaccine	(Kong et al., 2013; Fukuyama et al., 2015)
HA/DPNG	R: H or − COCH_3_ or **PEI**^(6)^	Paclitaxel	Treatment of heterogeneous tumor	(Yim et al., 2013)
PA/SDM	R: H or − CH_3_COCH_3_ or Succinylated sulfadimethoxine group	Doxorubicin	pH-responsive for targeted tumor	(Na & Bae, 2002; Na et al., 2003a)
PUL-DO/bHis	R: H or Deoxycholic acid group or N-Boc-l-histidine group	Doxorubicin	pH-responsive for targeted tumor	(Na et al., 2007)
acL-CHP	R: H or acid-labile group^(7)^	Bovine serum albumin	pH-sensitive for tunable release	(Morimoto et al., 2013)
CMP-PNIPAM	CMP: Carboxymethyl pullulan; **PNIPAM**^(8)^	Diphenhydramine hydrochloride	Thermo- and pH-responsive release	(Asmarandei et al., 2013)
PLP	R: H or Poly(l-lactide)^(9)^	Doxorubicin	Thermo-responsive for long-term release	(Seo et al., 2012)
SPL	R: H or Succinyl group or Poly(l-lactide)^(9)^	Lysozyme, etanercept	Thermo-responsive forlong-term release	(Jung et al., 2013, 2014)
SpP	R: H or Spiro form or Merocyanine form^(10)^	Citrate synthase, pyrene	Photo-responsive for protein delivery	(Hirakura et al., 2004; Wang et al., 2014)
PL/phA	R: H or **phA**^(11)^	–	Photodynamic therapy	(Bae et al., 2010)
PFP	R: H or phA or Folic acid group	–	Photodynamic therapy	(Bae & Na, 2010)
CHP-*o*-NB	R: H or ***o*-NB**^(12)^	Insulin	Patterned film	(Nishimura et al., 2016)
NIR-PNG	R: H or NG^(13)^	IRDye800	Lymph node mapping	(Noh et al., 2012; Kong et al., 2015)
Gd-CHPOA	R: H or Cholesteryl group or Acryloyl-moiety^(14)^; Gd-chelating crosslinker^(15)^	–	Tumor imaging	(Chan et al., 2015)
ImCHP	R: H or Cholesteryl group or Imidazolyl group^(16)^	–	Redox sensitivity	(Sasaki et al., 2011)
PulSTS-PNIPAM	R: H or **STS**^(17)^ or **PNIPAM**^(8)^	–	Thermo- and redox-responsive system	(Morimoto et al., 2008b,c)
PA	R: H or –COCH_3_	Clonazepam	Sustained release	(Jung et al., 2003)
BPA	R: H or –COCH_3_ or Vitamin H group	Doxorubicin	Targeted tumor	(Na et al., 2003b; Park et al., 2006)
PUL/DOCA	R: H or Deoxycholic acid group	Doxorubicin	Sustained release	(Na et al., 2006)
PUL/FA	R: H or Folic acid group	Doxorubicin	Anticancer	(Kim et al., 2008)
PUL/LLA	R: H or l-lactic acid oligomer^(18)^	Carmofur; Peostaglandin E_2_	Antitumor; Induced immunological tolerance	(Miyazaki & Tabata, 2009; Okamoto et al., 2011)
CAP	R: H or Modified carborane^(19)^	Carborane	Boron neutron capture therapy	(Kawasaki et al., 2017)

PEI: polyethyleneimine; PNIPAM: poly-*N*-isopropylacrylamide; phA: pheophorbides-A group; *o*-NB: *ortho*-Nitrobenzyl-substituted cholesteryl group; STS: sulfanylthiocarbonylsulfanyl group.

Important structures marked with consecutive numbers (6)–(19) are redrawn within the Supplemental File ‘Numbered Structures’.

Although CHPNH2 was toxic to cortical neurons, linkage with both monomeric and oligomeric β-amyloid (Aβ1-42) significantly reduced toxicity (Boridy et al., 2009). The enormous potential of nanogel scaffolds for protein encapsulation has been widely recognized. Loading BSA or β-galactosidase (β-Gal) helped CHPNH2 to form colloidally stable monodispersed nanoparticles (<50 nm) that are capable of effective transportation into HeLa cells even in the presence of serum (Ayame et al., 2008). Afterwards, Shimoda explored the mechanism of internalization into cells via clathrin-mediated endocytosis, specifically macropinocytosis and caveolae-mediated endocytosis. He noted that a portion of cationic nanogels escaped from the endosomes after a 24 h incubation (Shimoda et al., 2011). Just as important, the protein-loaded cCHP nanogels were able to functionally regulate cells even under the condition of low heparin sulfate expression. For example, staurosporine was able to induce apoptosis in J558L cells through reduced mitochondrial membrane potential, but the percentage of cells with normal mitochondrial membrane potential was sharply increased by treatment with a complex of the anti-apoptotic protein Bcl-xL and cCHP nanogel (Watanabe et al., 2011).

With the assistance of cationic liposomes, QDs have been applied in the long-term intracellular imaging field for a few years, but they readily formed aggregates of several hundred nanometers in diameter in the cytoplasm (Derfus et al., 2004). Therefore, scientists designed CHPNH2 to deliver QDs into cells. In the case of CHPNH2-QD hybrid nanoparticles, the fluorescence observed in normal cells, living cells, and cancer cells appeared to uniformly spread in the cytoplasm. Additionally, the fluorescence intensity was comparable with that in the cationic liposome (Hasegawa et al., 2005; Toita et al., 2008). It indicates that CHPNH2-QD hybrid nanoparticles may be a promising fluorescent probe for bioimaging and regenerative medicine.

Self-nanogels that load biomolecules such as proteins have been well studied because they are an attractive application for vaccines (Tahara & Akiyoshi, 2015; Kyogoku et al., 2016). Likewise, remarkable strides have been made in the creation of artificial vaccines based on cCHP nanogels (Yuki et al., 2013). The nanogel-based pneumococcal surface protein A (PspA) nasal vaccine (cCHP-PspA) was observed to induce PspA-specific Th17 and antibody responses against *Streptococcus pneumoniae* (Kong et al., 2013). *Clostridium botulinum* type-A neurotoxin (BoHc/A) released from cCHP nanogel without co-administration of mucosal adjuvant could simultaneously induce vigorously specific systemic humoral immunity, cellular immunity, and mucosal immunity. cCHP-BoHc/A continuously adhered to the nasal epidermal membrane and then was gradually released, and BoHc/A was taken up by mucosal dendritic cells. After exocytosis, BoHc/A was uptaken by CD11+, which triggered respiratory system immunity (Nochi et al., 2010). There was no deposition in the olfactory bulbs or brain after nasal administration in macaques (Fukuyama et al., 2015). These findings support a safe, effective, and practical strategy for mucosal immunity to infectious diseases in the respiratory tract.

**Figure 2.**
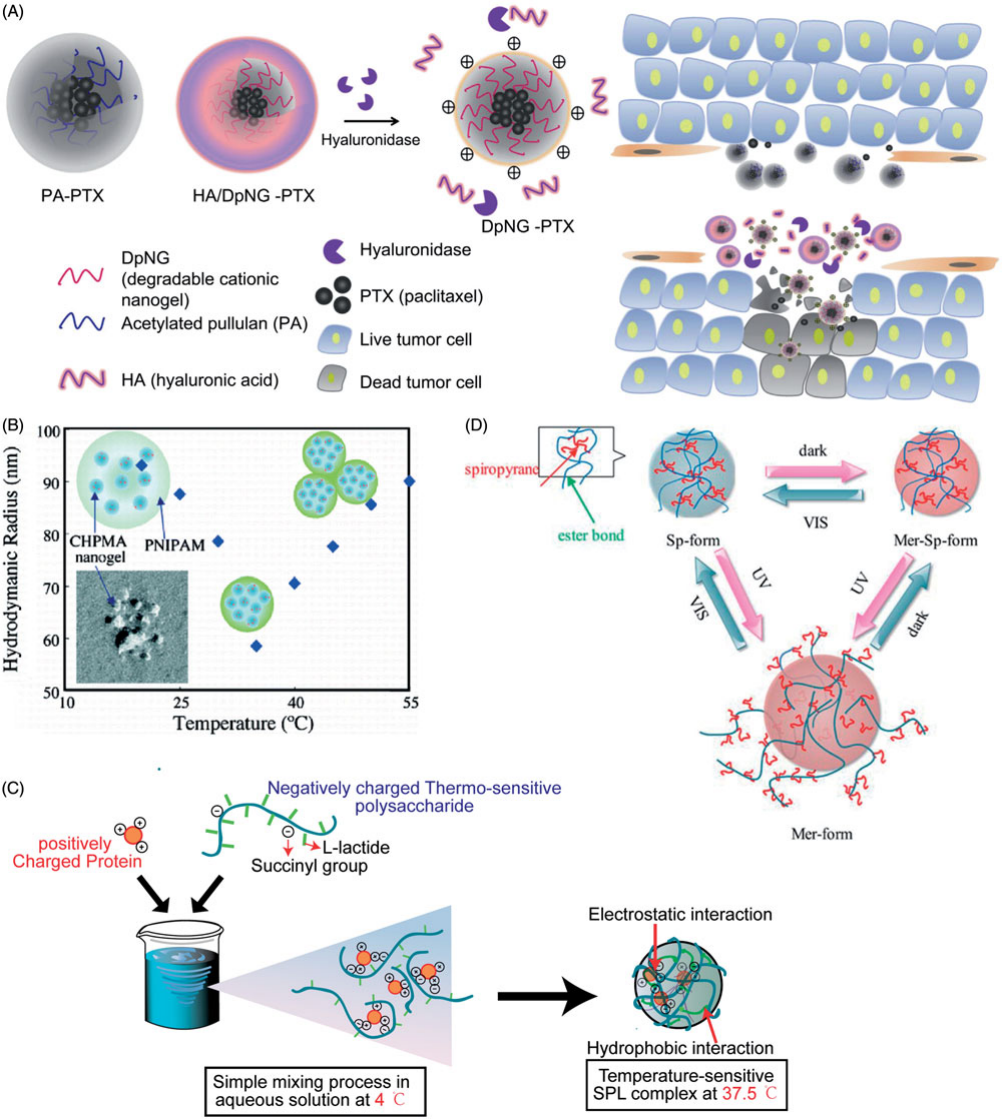
(A) Schematic illustration showing the composition of the degradable deep penetrating cationic nanoparticle (HA/DpNG-PTX, Table 3), and how it can be used to penetrate into tissue; (B) temperature-induced changes in the morphology and association of CHPMA-PNIPAM nanogels and SPL complexes; swollen nanogels formed by the association of a few CHPMA nanogels, shrunken nanogels, and botryoidal nanogels are shown; (C) aqueous condition at 4 °C but hydrogel condition at 37.5 °C is shown; (D) schematic illustration of SpS nanogel under UV–Vis light. HA/DpNG-PTX: hyaluronic acid-coated degradable cationic nanogel–paclitaxel; CHPMA-PNIPAM: methacryloyl-substituted CHP-poly-N-isopropylacrylamide; SPL: succinated pullulan-poly(l-lactide).

The tumor microenvironment with leaky vasculature, impaired lymphatic drainage, and a high density of cells as well as extracellular matrix are the biggest barriers that prevent nanoparticles from penetrating into interstitial tumor space (Hui & Chen, 2015). However, cationic nanogel may overcome such physical blocks via electrostatic interaction with negatively charged pores of the vessel wall (Stylianopoulos et al., 2013). Hyaluronic acid-coated degradable cationic nanogel (HA/DpNG-PTX, Figure 2(A) and Table 3) was responsible for the highest anticancer efficacy through a synergic effect between PTX and DpNG. It should be noted that most healthy tissues remain undamaged following treatment due to the reduced physical electrostatic pressure of cationic charge after degradation. At the high concentration, the cationic charge of DpNG disrupted the cell membrane and induced necrosis. The electrostatic interaction between DpNG and the plasma membrane absorbed the anionic charged lipid bilayer, which led to lateral phase separation via increased lipid flip/flop. At the low concentration, the DpNG-PTX was internalized into the cytosol through endocytosis, while apoptosis was induced by the PTX (Yim et al., 2013). Additionally, liver and kidney sections did not exhibit any abnormal signs when the backbone ((α-(1–4) or (α-(1–6) linkages in pullulan) of DpNG was degraded by hydrolysis either enzymatically or chemically under physiological conditions.

### pH-responsive

The measured acidic pH (approximately 6.5–7.2) of the extracellular fluid of solid tumors has extensively been documented for a few decades (Offerman et al., 2014). The endosomes and lysosomes in tumor cells are more acidic (pH approximately 5.0) as compared to physiological conditions. A mechanism has been developed whereby pH-induced anticancer drugs release from pullulan nanogels that are responsive to tumor acidic condition, and this could be an excellent mode for tumor targeting (Na & Bae, 2002; Na et al., 2003a,b, 2007; Morimoto et al., 2013).

A pH-responsive polymer hydrogel, pullulan acetate/sulfonamide (PA/SDM) (Table 3), exhibited good stability upon dilution and remained at pH 7.4 for over 10 days. However, PA/SDM nanogels shrank and aggregated below pH 7.4, and this would be significantly aggravated in the tumor microenvironment. The author assumed that the deionized SDM in the nanoparticles below pH 7.4 formed new hydrophobic domains, which perturbed the internal structure and led to conformational changes and the dehydration of the PA/SDM nanoparticles (Na & Bae, 2002). Furthermore, doxorubicin (DOX)-loaded PA/SDM exhibited higher cytotoxicity that may be attributed to the rapid release of DOX triggered by tumor pH as well as enhanced interaction with cells (Na et al., 2003a,b). Similarly, a research group introduced N-Boc-histidine (bHis) as a pH-sensitive moiety into pullulan-deoxycholic acid conjugate. PUL-DO/bHis (Table 3) nanogel accelerated the release of DOX via the deformation of the inner core, which was induced at pH values below 6.8. The author demonstrated that ionized imidazole in the gel at lower pH might create charge − charge repulsions between His-grafted chains or the swelling of the core by hydrophilization of the histidine part, resulting in interior structural changes of the PUL-DO/bHis nanogels (Na et al., 2007). Nobuyuki Morimoto prepared acid-labile CHP (acL-CHP) nanogels at neutral pH, derived from grafting vinyl ether-cholesterol groups onto a pullulan backbone. Size-exclusion chromatography (SEC) analysis demonstrated that these acL-CHP nanogels remain stable at physiological pH, but the grafts were nearly 80% degraded after 24 h at pH 4.0, whereas 18.2 ± 7.1% and 3.9 ± 0.9% free cholesterol were found in acL-CHP nanogel solutions that had been exposed to pH 4.0 and 7.4 for 24 h, respectively (Figure 1(D)). Surprisingly, the observed trapped-FITC-BSA release rate was slower than that of acL-CHP hydrolysis at pH 4.0, and the author hypothesized that BSA exhibited a reversible N-F transition via an intermediate form that participated in a stable complex with nanogels; the formation of acetal byproducts may be another reason for the slow release rate (Morimoto et al., 2013).

### Thermo-responsive

Among the internal and external stimuli, thermo-responsivity is one of the best signals for ensuring ease and safety in medical applications. Temperature-sensitive materials have been used to enhance the physical stability of proteins by rendering protein structures to be more rigid, which creates a controlled microenvironment around the protein (Shakya et al., 2010). Also, thermo-sensitive hydrogels can swell and de-swell, alternating in response to human body temperature changes (Asmarandei et al., 2013).

Poly-N-isopropylacrylamide (PNIPAM, Table 3), exhibiting a lower critical solution temperature (LCST) of 32 °C, is one of the most widely investigated thermo-responsive materials used for thermally triggered delivery (Berndt et al., 2006; Blackburn & Lyon, 2008). Furthermore, the phase transition temperature modestly depends on the structure, location along the chain, and level of incorporation of hydrophobic groups (Akiyoshi et al., 2000; Blackburn & Lyon, 2008; Morimoto et al., 2008a,b). As a result, a certain ratio of NIPAM grafted onto (or mixed with) pullulan has been extensively found in the past years (Morimoto et al., 2006, 2007, 2008a,b). For example, hydrogel releases 50% diphenhydramine hydrochloride in phosphate buffered saline (PBS, pH 7.4) in 0.5 h at 20 °C and in approximately 3.5 h at 37 °C (Asmarandei et al., 2013). Interestingly, CMP-PNIPAM (Table 3) nanogels of suitable composition exhibited two distinct volume transitions during heating/cooling cycles, and both steps in this temperature response were reversible (Morimoto et al., 2007). As shown in Figure 2(B), the hydrodynamic radius (*R*_h_) of nanogels reproducibly changed from approximately 70–60 nm in the 30–35 °C temperature domain, followed by a sharp increase in *R*_h_ from approximately 60–100 nm in a 35–50 °C window. The author postulated that the primary reason for these changes was that PNIPAM gels shrank as a consequence of increased hydrophobicity upon dehydration of the PNIPAM chains when heated through LCST of PNIPAM, and then the gels swelled upon further heating because of a growing aggregation number of associated nanogels by grafted PNIPAM chains (Akiyoshi et al., 2000; Morimoto et al., 2007). Additionally, the shrinking half-time of the gels was above 2 min, which might be useful in many fields where the release of substances such as hydrophobic drugs and proteins is desired (Morimoto et al., 2008a,b).

However, clinical application of PNIPAM in drug delivery systems is limited due to its nonbiodegradability. For this reason, Seo et al. applied l-lactide for polymerizing with pullulan, which resulted in a heat-related behavior (Seo et al., 2012; Jung et al., 2013, 2014). The particle size of poly(l-lactide) (PLP) (Table 3) nanogels with relatively higher lactide content enlarged rapidly over 32 °C. Moreover, the amount of DOX released within DOX-loaded PLP nanogels at 42 °C was more than that at 37 °C after 6 h, which indicated that more HeLa cells were killed at 42 °C, providing the potential application of hyperthermia therapy for solid tumors (Seo et al., 2012). Thermo-sensitive succinylated pullulan-g-oligo(l-lactide) (SPL) (Table 3) complexes were readily dissociated at 4 °C but remained stable at 37.5 °C via electrostatic attraction and hydrophobic interaction even in the presence of 150 mM salt and 10% serum. A schematic illustration of SPL complex is shown in Figure 2(C). Fluorescence images *in vivo* revealed that the signal intensity was apparent for longer (11 days) than that of the polyelectrolyte complex. It indicated that SPL complexes could maintain the bioactivity of encapsulated lysozyme *in vitro* while lysozyme was released from SPL complexes in a sustained manner *in vivo*. These results suggest the potential for long-term delivery of therapeutic proteins (Jung et al., 2014).

### Photo-responsive

Compared with other stimuli such as pH and temperature, light offers a milder activation, more precise remote spatiotemporal control, and easier use (Bertrand & Gohy, 2017). Light-sensitive nanogels can accurately deliver doses specific to individual patients through wavelength adjustments. This property would be beneficial for driving the accumulation of photodynamic agents in tumor sites by the enhanced permeability and retention (EPR) effect, thereby controlling photoactivity. Also, tumor imaging can be another useful application (Yue et al., 2017).

Spiropyrane-bearing pullulan (SpP) nanogels provide cross-linking points similar to CHP nanogels (Hirakura et al., 2004). The type of light (UV, Vis-, or dark) and irradiation time could affect the conversion among the amphiphilic Mer-Sp-form, hydrophobic Sp-form, and hydrophilic Mer-form (Figure 2(D) and Table 3) that controls the capacity of citrate synthase-trapped refolding activity (Hirakura et al., 2004) or pyrene-trapped release (Wang et al., 2014). The release amount and rate decreased in the following order: Mer-Sp-form > Mer-form > Sp-form. The author considered that the lowest release might be due to the hydrophobic core structure of the Sp-form. The second low release was attributed to the diffusion of proteins from the hydrophilic shell of the Mer-form structure when the Sp-form transferred into the Mer-form under UV irradiation, whereas the disappearance of electrostatic interaction during the transformation into the Mer-Sp-form may induce less interaction with proteins to promote the release from nanogels. Pullulan esterified with folic acid and pheophorbide-A (phA, Table 3) subsequently could prepare self-organized pentafluorophenyl (PFP) nanogels in water or PBS, which led to the self-quenching effect among phAs, but phA in nanogels restored photoactivity when co-incubated with HeLa cervical carcinoma cells. The author inferred that nanogels were internalized into the cancer cells by folate-mediator endocytosis and then degraded by various enzymes in the endosome and lysosome (Bae & Na, 2010; Bae et al., 2010;). Furthermore, photoactivity did not immediately appear but was detected after 30 min; a significant increase over 12 h was observed (Bae & Na, 2010). This may contribute to the possibility for photodynamic therapy of tumors with minimal unfavorable phototoxicity. Tomoki Nishimura constructed a photo-responsive cholesteryl pullulan (termed CHP-o-NB, as shown in Table 3) by linking ortho-nitrobenzyl that could be partly cleaved under the irradiation of 365 nm light, which resulted in the decomposition of nanogel-based materials at the masked site. This can be used for the preparation of protein patterning on nanogel-based film towards biochip (Nishimura et al., 2016).

Nanoprobes called near-infrared polymer nanogel (NIR-PNG), which are NHS-terminated IRDye800 (or IRDye900) conjugated with aminated CHP (NIR-PNG, Table 3), could dramatically enhance the photostability of dye. NIR-PNG nanoprobes (nearly 30 nm) were easily uptaken into the lymph system of animals, as discovered during experiments conducted by (Noh et al. (2012). The NIR-PNG signal was still detected in the canine stomach when evaluated one week after injection. This can be used as active molecular imaging probes for sentinel lymph node mapping, which will furnish potential utility in cancer surgery such as sentinel node navigation surgery in gastric cancer (Kong et al., 2015). Surprisingly, the presence of acryloyl groups on CHPOA nanogels allowed easy attachment of Gd-chelating cross-linkers (Table 3) through photoinitiation, endowing stable and longer-lasting tumor imaging. The clear tumor delineation could aid in accurate and precise tumor excision. Additionally, there was no toxicity observed in mice up to 3 months after injection of Gd-CHPOA (Chan et al., 2015).

### Redox-responsive

The redox potential gradient between normal cells and tumor cells has been extensively explored (Huo et al., 2014). Compared to normal cells, tumor cells contain a strongly reducing environment due to the overproduction of intracellular glutathione and reactive oxygen species (Luo et al., 2016). At present, redox-responsive pullulan nanogels are being intensively researched. Yoshihiro Sasaki et al., reported a novel redox-sensitive CHP (ImCHP) nanogel with redox couple Co(II) and Co(III) (Sasaki et al., 2011). When the labile Co(II) was oxidized to inert Co(III) by addition of 0.3% w/w H_2_O_2_, there was no obvious change in the MW and radius of ImCHP. This phenomenon indicated that ImCHP nanogels were crosslinked by coordinative interactions with Co(III) to form inter-nanogel crosslinking or intra-nanogel crosslinking. Even in the presence of β-CD, ImCHP nanogels with Co(III) were slightly swelled without dissociation. This suggested that ImCHP formed dual network nanogels with physically crosslinked and coordinative crosslinked points. Grafting sulfanylthiocarbonylsulfanyl (STS, Table 3) groups onto a pullulan framework (termed PulSTS) could produce easily oxidized thiols that are linked to the end of each PNIPAM chain. The PulSTS-PNIPAM nanogel exhibited dual stimuli-responsive parameters that may control the release of active agents entrapped in nanogel via simultaneous stimuli of temperature and redox (Morimoto et al., 2008a,b).

### Modified-release and targeting

Pullulan esterified with carboxylate could become a more stable carrier that can accept increased drug loading with larger encapsulation efficiency if there is an increase of the number of substituents such as deoxycholic acid (Na et al., 2006), folic acid (Kim et al., 2008), and polylactic acid (Miyazaki & Tabata, 2009; Okamoto et al., 2011). Drugs are released in a modified manner, and even an increased degree of acetylation of pullulan could decrease the release rate (Jung et al., 2003). Shimizu successfully incorporated recombinant murine interleukin-12 (IL-12) (rmIL-12) into CHP nanogel by co-incubation at room temperature. The rmIL-12 within the nanogel slowly released after subcutaneous injection into mice. However, the intravenous or intraperitoneal injection of CHP/rmIL-12 led to a rapid release of rmIL-12 due to frequent encounters with a high concentration of serum proteins such as BSA, as well as phagocytosis by hepatic and splenic macrophages (Shimizu et al., 2008). Afterwards, cross-linking of acrylate group-modified CHP nanogel (CHPANG) with PEGSH by Michael addition solved the problem described above (Hasegawa et al., 2009). Administration of W9, which is a bone resorption inhibitor peptide, at 24 mg/kg/day significantly prevented low calcium-induced reduction of bone mineral density in the long bones and lumbar vertebrae. An osteoclastogenesis inhibition assay *in vitro* confirmed the delayed release profile of W9 from hybrid hydrogels as compared to conventional CHP nanogels (Sato et al., 2015).

Targeting the desired site of action would not only enhance the therapeutic efficacy of drugs but also allow reductions in the dosage and undesirable side effects. Therefore, some researchers prepared targeted carriers on the basis of characteristics of tumor stroma (Hirakura et al., 2010). It has been demonstrated that the content of vitamin H in cancerous tumors is higher than that in normal tissue, and rapid proliferation of tumor cells may require extra vitamin H (biotin) (Chen et al., 2010). Na prepared biotinylated pullulan acetate (BPA), which was able to interact with HepG2 cells by vitamin-receptor recognition. The content and release rate of DOX encapsulated in the BPA nanoparticles decreased along with an increasing degree of vitamin H substitution due to a lower hydrophobicity (Na et al., 2003a,b; Park et al., 2006). High activity of hyaluronidase in tumors presents a unique tumor microenvironment (Benitez et al., 2011). Hyaluronic acid (HA)-coated cCHP was converted to uncoated cCHP by the hyaluronidase-mediated degradation of the HA coating. Consequently, the HA/DpNG-PTX-treated group exhibited significantly smaller tumor volumes compared with the control group treated with PTX (Yim et al., 2013). Recently, there has been some exploration of *in vivo* vaccine delivery of the CHP nanogel via ‘immunological stealth mode’. For example, CHP nanogel can efficiently travel to a draining lymph node due to the small size and uncharged surface after subcutaneous injection. The CHP nanogel was preferentially engulfed by medullary macrophages but not sensed by other macrophages and dendritic cells. As a result, nanogel-based vaccine significantly inhibited *in vivo* tumor growth in the prophylactic and therapeutic settings, and this new strategy can be used to enhance vaccine efficacy (Muraoka et al., 2014).

## Discussion

From the description above, we know that self-nanogels based on pullulan derivatives could carry drugs and/or proteins. As the schematic diagram described in Figure 1(D) shows, abundant distinctive scaffolds of pullulan nanogel can be conceived, including the incorporation of environmentally responsive groups, controlling the change of surface charge and morphology, and coupling or chelating the metallic elements. Interestingly, CHP nanogel itself was found to effectively accelerate wound healing (Kobayashi et al., 2009; Miyahara et al., 2012). Additionally, nanogel crosslinked hydrogel promoted the rate and extent of wound healing when it was combined with a silicone sheet (Maeda et al., 2017). When CHP nanogels carried tumor necrosis factor (TNF)-α, there was a minimization of the influence of inflammatory cytokines other than TNF-α (Nagano et al., 2011). Importantly, protein cancer vaccines based on pullulan nanogels have made great progress (Tahara & Akiyoshi, 2015). After complexing with CHP nanogels, immunogenic proteins including HER2, NY-ESO-1, and MAGE-A4 induced various immune responses at different levels, as shown in Table 4. Furthermore, the vaccines mainly induced CD4 T-cell response, CD8 T-cell response, and antigenic protein-related antibody responses. Relevant clinical trials are primarily in phase I and are progressing with safe and well-tolerated vaccines (Kitano et al., 2006; Aoki et al., 2009; Saito et al., 2014).

**Table 4.** Clinical studies of CHP nanogels.

Loaded objects	Cancer type and clinical cases	References
HER2	Solid tumors; well tolerated, CD4^+^ T-cell response in 5/9 patients, CD8^+^ T-cell responses in 4/9 patients	(Kitano et al., 2006)
HER2 plus GM-CSF	146HER2-specific IgG antibody response in 14 patients; fastly reached plateau levels vaccinated with CHP-HER2 plus GM-CSF	(Kageyama et al., 2008)
NY-ESO-1	NY-ESO-1 antibody response in all 9 patients	(Kawabata et al., 2007)
	CD4^+^ T-cell response in 7 including 3 esophageal cancer, 1 malignant melanoma and 3 prostate cancer of 9 patients	(Uenaka et al., 2007)
	Melanoma cell; CD4^+^ and CD8^+^ T-cell responses, detecting CD4^+^CD25^+^ Foxp3^+^ Tregs and CD68^+^ immunoregulatory macrophages	(Tsuji et al., 2008)
	Esophageal cancer; antibody, CD4^+^ and CD8^+^ T-cell responses in 7,7 and 6 of 8 patients	(Wada et al., 2008)
	Lung adenocarcinoma; antibody, CD4^+^ and CD8^+^ T-cell responses	(Isobe et al., 2009)
	Non-small cell lung cancer; spontaneously remission for one patient	(Nakamura et al., 2009)
	Esophageal and prostate cancer; antibody responses in 8/9 patients	(Kawada et al., 2012)
	Advanced/metastatic esophageal cancer; 100-μg cohort inducing antibody responses in 3/13 patients and 200-μg cohort in 7/12 patients	(Kageyama et al., 2013)
NY-ESO-1 plus HER2	Esophageal cancer; comparable antibody response with CHP/NY-ESO-1 and CHP/HER2	(Aoki et al., 2009)
MAGE-A4	Advanced esophageal, stomach or lung cancer; MAGE-A4-specific humoral immune response in 4/20 patients, CD4^+^ and CD8^+^ T-cell responses in 3/20 and 6/20 patients	(Saito et al., 2014)
MAGE-A4	5 colon, 1 esophageal, 1 papilla of Vater, 1 breast, 1 pancreatic cancer; IgG response in 7/9 patients, IgE response in 4/7 patients	(Kyogoku et al., 2016)

Despite the benefits of nanogels, we have to acknowledge that there are some important issues to be considered. First, safety is still the primary problem. Although molecule-encapsulated nanogels formed by hydrophobic interactions of physical cross-linking are feasible in lab tests, unpredictable toxicity and undiscovered side effects still exist. It has been reported that drug-encapsulated nanogel may interact with normal cells and change their normal functions (Na et al., 2003a,b). The strong cation-based material exhibits several undesirable characteristics, including the aggregation of erythrocytes and platelets (Yim et al., 2013). CHPNH2 nanogels are toxic to the central nervous system, particularly in primary cortical cultures (Boridy et al., 2009). Accurate targeting of the delivery may be one effective method to reduce toxicity. For instance, PGE2/CHP induced new bone formation only at the site of injection instead of at distant sites of the skeleton (Kato et al., 2007). Similarly, carborane-bearing pullulan (CAP, Table 3) formed stable self-nanogel through hydrophobic interactions with carborane. Compared to conventional boron neutron capture therapy, the CAP nanogels successfully reduced the cytotoxicity of carborane, were internalized into tumor cells by endocytosis, acted as a dual-delivery mode of therapy, and accumulated in tumors *in vivo* (Kawasaki et al., 2017).

Second, as previously mentioned, many protein-loaded CHP nanogels are prepared by dialysis. The procedure is quite time-consuming, and activities of encapsulated drugs may be influenced as well. However, hybrid hydrogel loads drugs with a simple mixing method that obtains higher encapsulated efficiency (Hasegawa et al., 2009), although hybrid hydrogels are not suitable for intravenous administration because of their size of several hundred nanometers. On the contrary, they are well suited to tissue engineering, including bone regeneration (Kato et al., 2007; Fujioka-Kobayashi et al., 2012; Yokota et al., 2014; Sato et al., 2015). Third, low encapsulation efficiency and low levels of drug loading are other two vital problems that must be solved for pullulan nanogels if they are to accommodate materials as efficiently as other polymer loading systems (Na et al., 2003a,b, 2006; Wang et al., 2014). Efficient therapeutic effects cannot be guaranteed when low concentrations of the desired substance are used. Fourth, CHP nanogels are only effective for a relatively short period, with limited long-term protein delivery. For example, vaccination with CHP-NY-ESO-1 did not prevent tumor growth in esophageal cancer patients (Wada et al., 2008). Similarly, lung adenocarcinoma restarted to progress after three years of vaccination with CHP-NYESO-1 (Isobe et al., 2009). Because of numerous problems, every effort has been made to improve the processes including development of hybrid hydrogel and combinations of various nanostructures. For example, CHPOA nanogels and gelatin hybrid fibers were constructed as a new platform for stable encapsulation of proteins. The potential advantages compared with hybrid hydrogels are that fibers can be preserved for a long time (Shimoda et al., 2016).

Last but not least, there is some evidence that cholesterol-modified hydrogels increased cell adhesion and proliferation (Kubinova et al., 2009). This result reminds us that cholesterol is useful for tissue engineering but it may not be suitable for delivery of antitumor agents. It was reported that docetaxel *in vivo* was degraded into stereoisomer 7-epidocetaxel, which may cause severe toxicity (Manjappa et al., 2013; Luo et al., 2017). Hence, as a chiral molecule, cholesterol might elicit isomerization and further influence the bioactivity of loaded drugs *in vivo*. These issues require further studies if additional approvals for clinical applications are to be obtained.

## Conclusions

Many investigations have revealed that pullulan nanogel is a facile and efficacious delivery system. Thus far, there has been significant progress with pullulan nanogel in wide fields including protein delivery, vaccine development, cancer chemotherapy, tissue engineering, and biological imaging. Significantly, pullulan can be modified by various functional groups in a simple method of synthesis and thereby express desirable activities in response to environmental factors such as pH, heat, light, and redox, to achieve satisfying objectives at specific sites. At the same time, this method may maintain the intrinsic or more strongly physicochemical properties of loaded agents than that of chemical modifications such as covalent bonding. In particular, the molecular chaperone-like activity of pullulan nanogel enables the possibility of achieving potent intracellular delivery.

Indeed, precision medicine will be the crucial window in future research. The development of carriers should be associated with the idea of ‘quality by design’, and tailor-made functional nanogels should be viewed as novel intelligent nanobiomaterials. More efficient targeting therapy and synergistic effects, multiple responses, and co-administration of various molecules, especially gene-based, should be considered for pullulan nanogel as the next focus of study. At present, there has been some progress on aspects of the polysaccharide nanogel gene delivery system investigated. However, clinical trials are still in phase I or II only for cancer vaccines, indicating that more preclinical studies are needed to provide convincing resources for supporting advanced clinical applications. We believe that there are promising potential applications for pullulan nanogel systems.

## Supplementary Material

IDRD_Liu_et_al_Supplemental_Content.pdf
