# Supplementary material for: Research progress of self-assembled nanogel and hybrid hydrogel systems based on pullulan derivatives
Source: Drug Deliv. 2018 Jan 15;25(1):278–92. doi: 10.1080/10717544.2018.1425776 (PMC6058595; doi:10.1080/10717544.2018.1425776)
Supplement: IDRD_Liu_et_al_Supplemental_Content.pdf [file IDRD_A_1425776_SM3605.pdf]

(1)

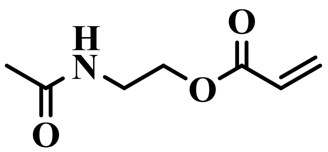

AOI: 2-(acryloyloxy)ethyl isocyanate

(2)

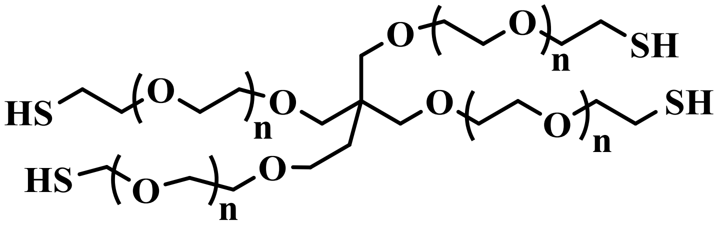

PEGSH: Pentaerythritol tetra (mercaptoethyl)polyoxyethylene

(3)

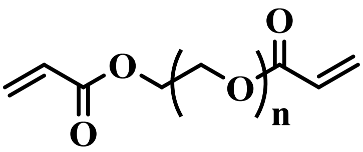

PEGDA: Polyrthylene glycol diacrylate

(4)

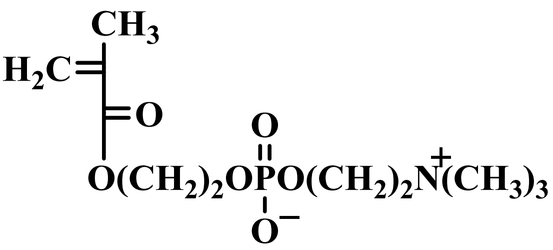

MPC: 2-methacryloyloxyethyl phosphorylcholine

(5)

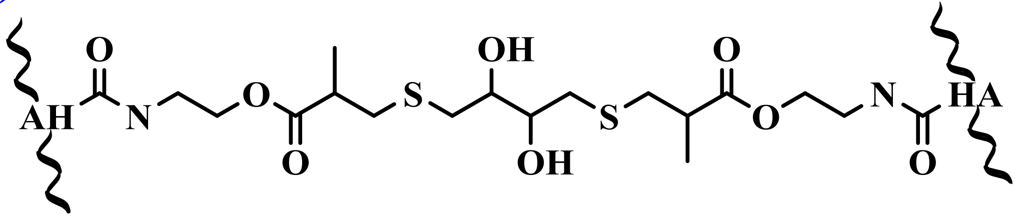

HA: Chemically cross-linked Haluronan

(6)

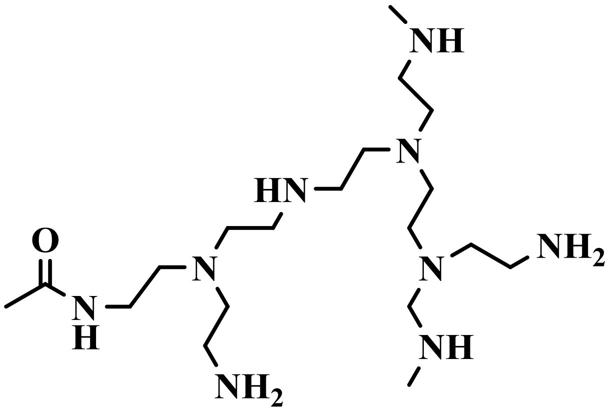

PEI: Polyethyleneimine group

(7)

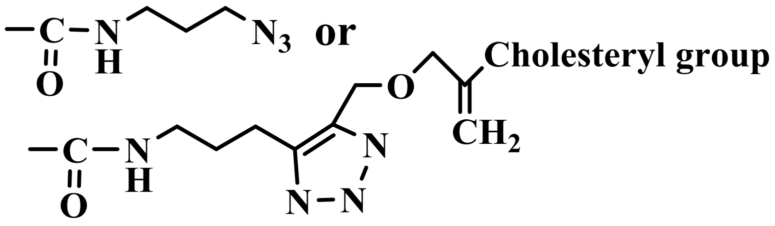

Acid-labile group

(8)

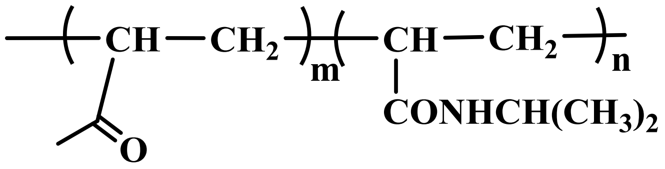

PNIPAM: Poly-*N*-isopropylacrylamide

(9)

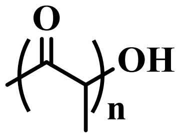

Poly (L-lactide)

(10)

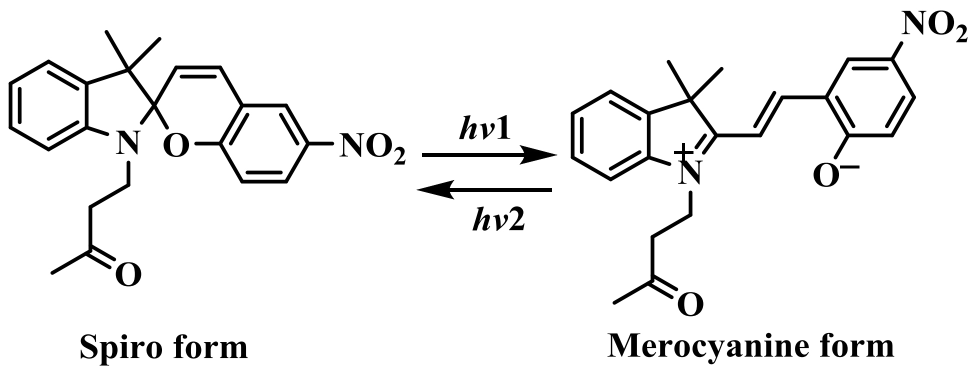

(11)

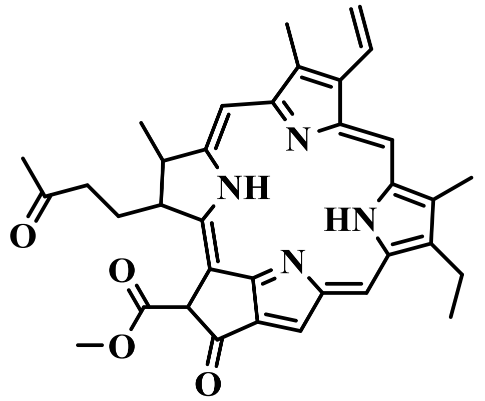

phA: Pheophorbides-A group

(12)

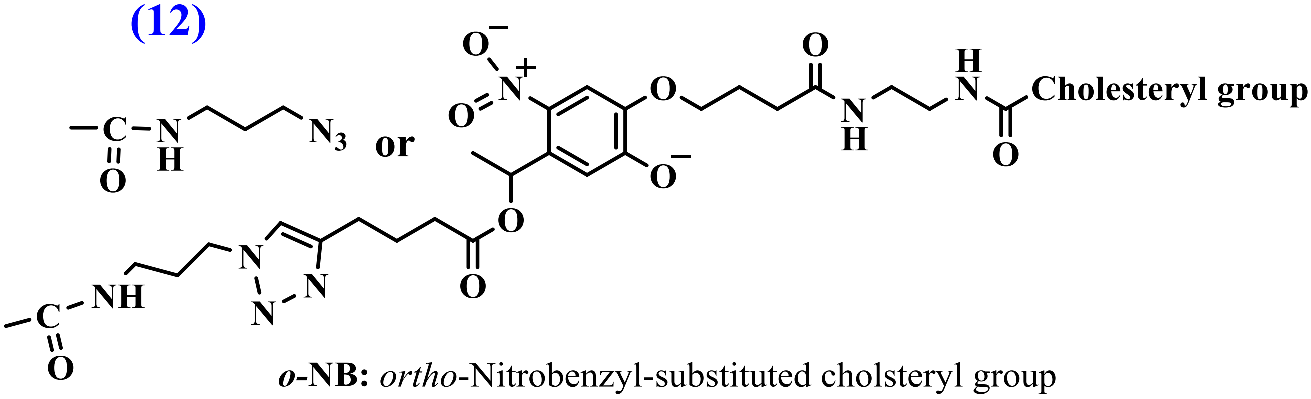

***o*-NB:** *ortho*-Nitrobenzyl-substituted cholsteryl group

(13)

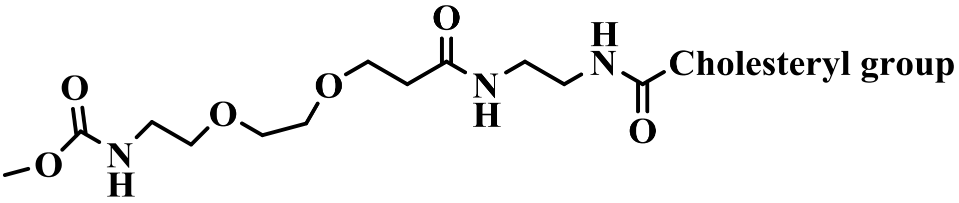

NG

(14)

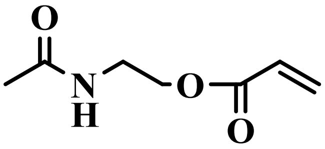

Acryloyl-moiety

(15)

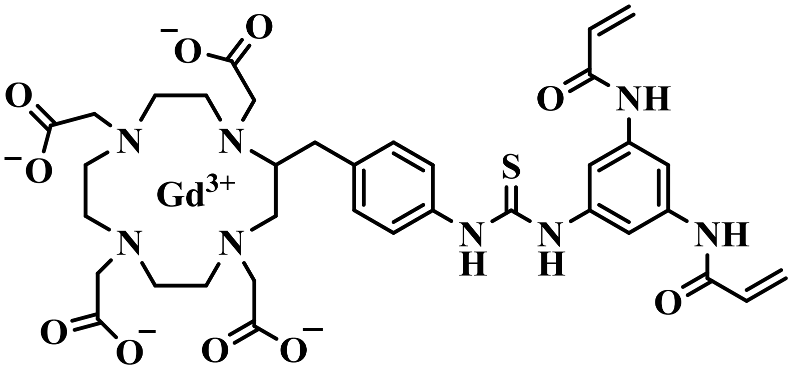

Gd-chelating crosslinker

(16)

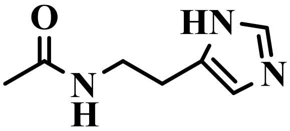

Imidazolyl group

(17)

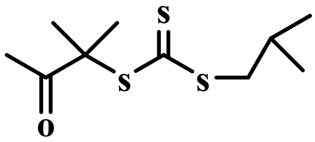

STS: Sulfanylthiocarbonylsulfanyl group

(18)

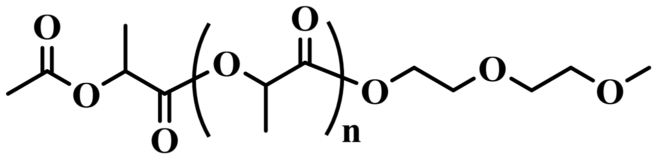

L-lactic acid oligomer

(19)

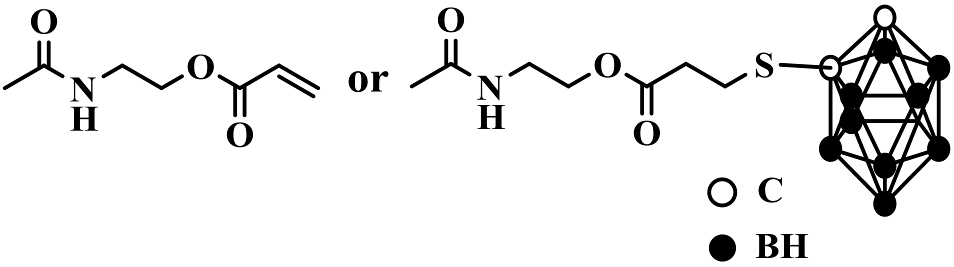

Modified carborane
